# Prevalence, Discontinuation Rate, and Risk Factors for Severe Local Site Reactions with Topical Field Treatment Options for Actinic Keratosis of the Face and Scalp

**DOI:** 10.3390/medicina55040092

**Published:** 2019-04-04

**Authors:** Alise Balcere, Māra Rone Kupfere, Ingrīda Čēma, Angelika Krūmiņa

**Affiliations:** 1 Department of Infectiology and Dermatology, Riga Stradiņš University, LV-1006 Riga, Latvia; dr.mararonekupfere@gmail.com (M.R.K.); Angelika.Krumina@rsu.lv (A.K.); 2 Department of Oral Medicine, Riga Stradiņš University, LV-1007 Riga, Latvia; Ingrida.Cema@rsu.lv

**Keywords:** actinic keratosis, topical therapy, severe local site reactions, risk factors, review

## Abstract

Actinic keratoses (AKs) are common lesions on chronically sun damaged skin, which are morphologically characterized by lower third to full thickness atypia of epidermal keratinocytes. These lesions carry a risk of progression towards invasive squamous cell carcinoma (SCC); therefore, treatment of visible lesions and the field in case of field cancerization is recommended. Treatment of AK includes the destruction of atypical keratinocytes that clinically presents with various degrees of erythema, scaling, crusting, erosion, and other visible and subjective symptoms. Such inflammatory reactions may have an impact on the patient’s social life and have shown to decrease compliance and adherence to therapy. Additionally, as various topical treatments have been proven to be effective in treating AK, tolerability of local site reactions (LSRs) might drive the decision for appropriate treatment in an individual scenario. Therefore, we aimed to review prevalence of severe LSRs among various topical treatments for AK. In addition, we summarized discontinuation rates due to LSRs and possible therapy-unrelated risk factors for the development of LSRs with increased severity.

## 1. Introduction

Actinic keratosis (AK) is one of the most common dermatological complaints in fair skinned individuals that represents the cumulative UV damage of epidermal keratinocytes. On clinical examination, AKs are variably thick and erythematous, poorly demarcated, and sometimes pigmented lesions on chronically sun-exposed skin [1]. Prevalence steadily increases with age and a recent Swiss study found that AK occurred in 25.3% of outpatients in general practice [2]. The clinical significance of AKs relies on the associated discomfort, cosmetic burden, and the possibility of progression to invasive squamous cell carcinoma (SCC). The traditional view considers that progression towards invasive SCC requires full thickness epidermal atypia, which is clinically usually characterized by thick and obvious lesions [3,4]. Nevertheless, a more recent study has shown that AK grade I, morphologically characterized by atypical keratinocytes in the lower third of the epidermis, is the most common type of AK overlying cutaneous invasive SCC [5]. This finding supports the understanding that all AKs require treatment. However, treatment is targeted towards the destruction of atypical keratinocytes, resulting in temporary inflammatory reactions with varying severity. Discussing the treatment-associated local site reactions (LSRs) before therapy may lead to delay in treatment initiation. During treatment, such reactions cause distress [6] and impact health-related quality of life [7]. A study by Strohal et al. [8] showed that 19.4% of patients treated with imiquimod 5% cream, scheduled unplanned visits due to their concern of LSRs. Moreover, LSRs lead to nonadherence, thus possibly reducing treatment efficacy [9]. As topical therapy is easy to use and a highly effective treatment modality for AK, we aimed to systematically search the PubMed database to review prevalence of severe LSRs among various topical treatments for AK. In addition, we summarized discontinuation rates due to LSRs and possible therapy-unrelated risk factors for the development of LSRs with increased severity. We did not review studies involving special equipment as conventional photodynamic therapy. For this review, “LSRs” included visual parameters such as, but not limited to, erythema, oedema, and erosions, and patient-reported subjective symptoms, such as pain and itching.

## 2. Severe Local Site Reactions with Topical Field Treatment for Actinic Keratosis of the Face and Scalp

### 2.1. Prevalence of Severe Local Site Reactions

The prevalence of severe LSRs among various topical field treatment options is summarized in Table 1 [10,11,12,13,14,15,16,17,18,19,20,21,22,23,24,25,26,27,28]. 

**Imiquimod.** Among all topical therapies, the highest prevalence of severe LSRs was reported in studies with imiquimod 5% [24] and imiquimod 3.75% [14], with treatment regimens not commonly used. The suggested treatment regimen for imiquimod 5% is three times per week for four weeks followed by four weeks of rest and a second cycle if needed. Such a regimen was used in three of the included studies, and reported the highest values of 31.0% [27] and 31.8% [26] for prevalence of severe erythema and 8.8% prevalence of severe scabbing or crusting [25]. In the last study by Rivers et al. [25], severe erythema was noted in 5% of patients. Such less frequent prevalence is probably due to study methodology as patient assessment was performed at baseline and at week 8. Comparatively lower 25.2% prevalence of severe erythema and 33.8% prevalence of any severe LSR was reported by Swanson et al. [13] with imiquimod 3.75% in two identical studies randomized to placebo.

**Ingenol mebutate.** Studies with ingenol mebutate gel 0.015% (IngMeb) mostly use composite scores to assess severity of LSRs. Each of six LSR parameters—erythema, flaking/scaling, crusting, swelling, vesiculation/pustulation, and erosion/ulceration is graded from 0 to 4, giving a maximum composite score of 24 [15,29]. Jim On et al. [16] reported data of two multicenter, randomized, parallel-group, double-blind, vehicle-controlled studies, involving face and scalp and classified patients with a composite score of 12 or higher as having a severe LSR. Thus, their reported prevalence of severe LSRs at day 4 was 24.5%. Skroza et al. [15] included 130 patients and assessed LSRs at day 3. The reported mean total composite scores did not allow to assess overall prevalence of severe reactions, but they reported grade 4 swelling in 17.7% of patients. Additionally, a study by Ricci et al. [30] reported severe pain and itching in 20.5% of treatment cycles with IngMeb. This was not included in the chart as prevalence was calculated from treatment cycles, not patients.

**5-fluorouracil.** Since its approval in 1970, topical 5-fluorouracil (5-FU) has become a well-established treatment for AK and LSRs are expected [31,32]. Three formulations − 5-FU 5%, 5-FU 0.5% and 5-FU 0.5% in combination with a salicylic acid 10% solution (5-FU/SA) are commercially available. A prospective, open-label, multicenter study by Stough et al. [18] included 277 patients treated with once daily application of 5-FU 0.5% cream for up to 4 weeks. Interim results of the face and scalp showed that severe LSRs developed in 19.1% of patients. Two studies assessed efficacy and safety of 5-FU/SA. In the first by Stockfleth et al. [10], in a randomized, placebo-controlled, double-blind, multicenter trial conducted in Germany, low dose 5-FU/SA was compared with 3% diclofenac in 2.5% hyaluronic acid gel (diclofenac HA). General disorders and administration-site conditions of severe intensity were reported in 27.8% of patients in the 5-FU/SA group and in 11.9% of patients in the diclofenac HA group. The second study by Simon et al. [20] compared 5-FU/SA with cryotherapy and included 33 patients in each treatment arm. Severe application site reactions were reported in six patients (18.2%) in 5-FU/SA arm and in one (3%) patient receiving cryotherapy.

**Diclofenac HA.** Pflugfelder et al. [19] reported 13.6%, which is the highest prevalence of severe LSRs in treatment with diclofenac HA. In a multicenter, randomized, open-label study with 418 included patients, they compared three-month vs. six-month treatment of actinic keratoses with diclofenac HA. Severe LSRs developed mainly in the first weeks of treatment and longer treatment only slightly increased the mean intensities of LSRs [19]. As already mentioned above, Stockfleth et al. [10] in a randomized, placebo-controlled, double-blind, multicenter trial conducted in Germany, reported general disorders and administration-site conditions of severe intensity in 11.9% of patients in diclofenac HA group.

**Daylight photodynamic therapy.** Only a single study reported a severe LSR in treatment with daylight photodynamic therapy (DL-PDT). This was a prospective, observational study conducted in Australia by See et al. [22], and they reported a single patient with severe post-treatment phototoxic reaction with erythema, pain, pruritus, and a skin burning sensation. This allowed a calculation of 1.2% of severe LSRs. Other studies on DL-PDT reported that none of the treated patients experienced severe LSRs [11,21,23].

### 2.2. Treatment Discontinuation Due to Local Site Reactions

**Figure 1:**
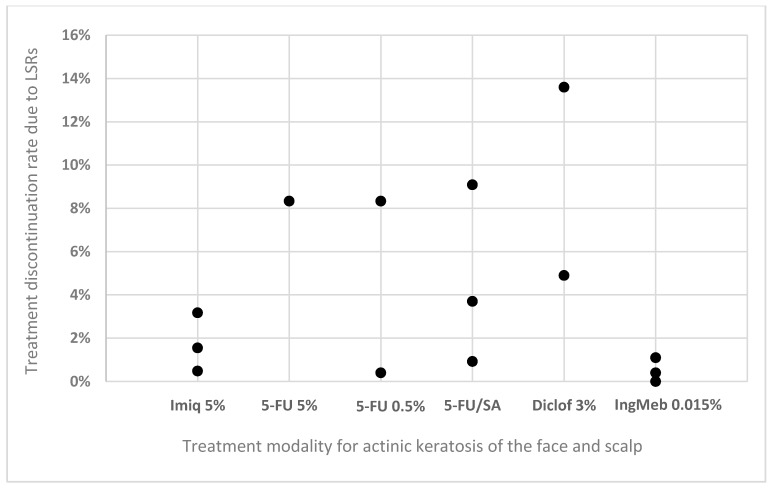
Reported rates of treatment discontinuation due to LSRs. Imiq 5%—imiquimod 5% cream; 5-FU 5%—5-fluorouracil 5% cream; 5-FU 0.5%—5-fluorouracil 0.5% cream; 5-FU/SA—5-fluorouracil in combination with salicylic acid 10% solution; Diclof 3%—3% diclofenac in 2.5% hyaluronic acid gel; IngMeb 0.015%—ingenol mebutate 0.015% gel.

In total, 14 articles reported treatment discontinuation rates due to LSRs with commonly used therapeutic regimens (Figure 1). The highest discontinuation rate of 13.6% was reported in a study by Pflugfelder et al. [19] that compared efficacy, tolerability, and quality of life of diclofenac HA used twice daily for three or six months. The study included 418 patients and eczematous reactions leading to discontinuation developed mainly in the first weeks of treatment. The second study with twice daily application of diclofenac HA was conducted by Stockfleth et al. [10] and reported a lower 4.9% discontinuation rate due to LSRs. Albeit, in this study, in the case of severe LSRs, application frequency was allowed to be reduced to once daily. The second highest rate of discontinuation due to LSRs was reported with 5-FU/SA by Simon et al. [20]. In the study evaluating efficacy, tolerability, and safety of low-dose 5-FU/SA topical solution vs. cryosurgery, they reported 9.1% discontinuation in the 5-FU/SA arm of 33 patients. As cryotherapy is a one-off treatment, it cannot be used as a true discontinuation comparator. Two other studies with 5-FU/SA and larger sample size reported 0.9% and 3.7% discontinuation rates [30,33]. Two studies reported discontinuation rates with low dose 5-FU cream. A study by Smith et al. [34] had 12 patients in the 5-FU 0.5% cream group, and the discontinuation of a single patient allowed a calculation of an 8.3% discontinuation rate. A study by Stough et al. [18] had 277 patients included and reported a discontinuation rate of 0.4%. A 5% 5-FU cream was used in one of the identified studies and included 50 patients. No discontinuations were reported [35]. Three articles showed discontinuation rates ranging from 0.5% to 3.2% with imiquimod 5% cream [25,27,36]. A discontinuation rate of up to 1.1% was reported in the studies with IngMeb [17,35,37]. This highest value was due to severe erosions in one of 88 included patients [37]. Treatment discontinuation due to LSR is not a concern for DL-PDT [11,21,22,23,38].

### 2.3. Therapy-Unrelated Risk Factors for Development of Local Site Reactions with Increased Severity

Severity of LSRs depends on the administered active ingredient and application frequency. Additionally, several studies have implications on patient-associated and environmental risk factors. Primarily, light pigmentation is a risk factor for both AK and treatment-induced severity of LSRs [39]. A study with IngMeb by Ricci et al. [37] with a standard patient-applied regimen of once daily IngMeb for three consecutive days, showed that patients with a fair skin type (phototype I–II) had stronger LSRs at day 4 and more erosions than patients with phototype III–IV. Moreover, although DL-PDT generally does not cause severe LSRs, in a study by Galvão et al. [40], moderate erythema after two hours of outdoor exposure was seen only in an albino patient. Other proposed risk factors for greater severity of LSRs with IngMeb are: the female gender, an age below 70 years, and Korean patients [41]. This last observation was suggested to be related to the difference of race and skin thickness [42]. The importance of environmental factors has been suggested in a study by Fargnoli et al. conducted in Italy from September to October. In particular, high outdoor temperature was associated with severity of LSRs and treatment efficacy of DL-PDT [38].

## 3. Conclusions

Local site reactions of severe intensity seem to be extremely common among topical therapies for AK, especially with imiquimod. The only therapeutic modality with low prevalence of severe LSRs is DL-PDT. Treatment discontinuation due to LSRs is also common, although the highest prevalence of treatment discontinuation due to LSRs is reported in studies with the longest treatment regimens, as with diclofenac, and not in studies reporting the highest prevalence rates of severe LSRs. Several patient-associated risk factors for the development of severe LSRs have been identified in studies with DL-PDT and IngMeb. Nevertheless, to have better evidence of individual risk for severe LSRs, further studies could identify more risk factors, and include other therapies.

## Figures and Tables

**Table 1:** Comparison of local site reaction (LSR) severity among various field treatment modalities.

Author	Year of Publication	Treatment Modality	Number of Patients	Application Frequency	Overall Prevalence of LSR	Prevalence of Severe LSR	Prevalence of Systemic Symptoms
Sotiriou et al. [21]	2018	DL-PDT with MAL	46	Once	71.6%	0%	0%
Sotiriou et al. [11]	2017	DL-PDT with MAL	26	Once	69.2%	0%	0%
See et al. [22]	2017	DL-PDT with MAL	81	Once	44.4% * (erythema)	1.2%	0%
Rubel et al. [23]	2014	DL-PDT with MAL	100	Once	39.0%	0%	0%
Serra-Guillén et al. [24]	2017	Imiquimod 5%	65	Once daily for 12 consecutive days	100%	58.5%	20%
Rivers et al. [25]	2008	Imiquimod 5%	42	Three times per week for 4 weeks followed by 4 weeks of rest and second cycle if needed	95.2% * (erythema)	8.8% * (scabbing or crusting)	14% * (upper respiratory infection)
Stockfleth et al. [26]	2007	Imiquimod 5%	828	Three times per week for 4 weeks followed by 4 weeks of rest and second cycle if needed	NR	31.8% * (erythema)	6% * (headache)
Alomar et al. [27]	2007	Imiquimod 5%	129	Three times per week for 4 weeks followed by 4 weeks of rest and second cycle if needed	96.9% * (erythema)	31.0% * (erythema)	NR
Korman et al. [28]	2005	Imiquimod 5%	241	Three times per week for 16 weeks	98.3% * (erythema)	33.2% * (erythema)	NR
Lebwohl et al. [12]	2004	Imiquimod 5%	215	Twice per week for 16 weeks	97.2% * (erythema)	17.7% * (erythema)	NR
Swanson et al. [13]	2010	Imiquimod 3.75%	160	Once daily 2-week on/off/on cycle	Almost all	33.8%	NR
Hanke et al. [14]	2010	Imiquimod 3.75%	162	Once daily 3-week on/off/on cycle	Almost all	54.9%	8% * (Influenza-like illness)
Skroza et al. [15]	2017	Ingenol mebutate gel 0.015%	130	Once daily for 3 consecutive days	100%	17.7% * (edema)	0%
Jim On et al. [16]	2016	Ingenol mebutate gel 0.015%	274	Once daily for 3 consecutive days,	100%	24.5%	NR
Pflugfelder et al. [19]	2012	3% diclofenac in 2.5% hyaluronic acid gel	418	Twice daily for 3 or 6 months	NR	13.6%	NR
Stockfleth et al. [10]	2011	3% diclofenac in 2.5% hyaluronic acid gel	185	Twice daily for up to 12 weeks	62.7%	11.9% **	
Simon et al. [20]	2015	5-fluorouracil 0.5%⁄salicylic acid 10.0%	33	Once daily for up to 6 weeks	8.8% * (erythema)	18.2%	NR
Stockfleth et al. [10]	2011	5-fluorouracil 0.5%⁄salicylic acid 10.0%	187	Once daily for up to 12 weeks	92.0%	27.8% **	
Stough et al. [18]	2008	5-fluorouracil 0.5%	277	Once daily for up to 4 weeks	87.0%	19.1%	NR

NR—not reported; DL-PDT—daylight photodynamic therapy; MAL—methyl aminolevulinate; * Prevalence of the highest symptom reported; ** Combined data of severe symptoms.
